# A Link between Virulence and Homeostatic Responses to Hypoxia during Infection by the Human Fungal Pathogen Cryptococcus neoformans

**DOI:** 10.1371/journal.ppat.0030022

**Published:** 2007-02-23

**Authors:** Cheryl D Chun, Oliver W Liu, Hiten D Madhani

**Affiliations:** Department of Biochemistry and Biophysics, University of California San Francisco, San Francisco, California, United States of America; David Geffen School of Medicine, United States of America

## Abstract

Fungal pathogens of humans require molecular oxygen for several essential biochemical reactions, yet virtually nothing is known about how they adapt to the relatively hypoxic environment of infected tissues. We isolated mutants defective in growth under hypoxic conditions, but normal for growth in normoxic conditions, in *Cryptococcus neoformans,* the most common cause of fungal meningitis. Two regulatory pathways were identified: one homologous to the mammalian sterol-response element binding protein (SREBP) cholesterol biosynthesis regulatory pathway, and the other a two-component-like pathway involving a fungal-specific hybrid histidine kinase family member, Tco1. We show that cleavage of the SREBP precursor homolog Sre1—which is predicted to release its DNA-binding domain from the membrane—occurs in response to hypoxia, and that Sre1 is required for hypoxic induction of genes encoding for oxygen-dependent enzymes involved in ergosterol synthesis. Importantly, mutants in either the SREBP pathway or the Tco1 pathway display defects in their ability to proliferate in host tissues and to cause disease in infected mice, linking for the first time to our knowledge hypoxic adaptation and pathogenesis by a eukaryotic aerobe. SREBP pathway mutants were found to be a hundred times more sensitive than wild-type to fluconazole, a widely used antifungal agent that inhibits ergosterol synthesis, suggesting that inhibitors of SREBP processing could substantially enhance the potency of current therapies.

## Author Summary

Opportunistic environmental pathogens adapt to hostile conditions within the host to cause disease. We describe two pathways in the pathogenic fungus Cryptococcus neoformans that are both necessary for adaptation to hypoxia and required for its virulence. One pathway uses a pathway homologous to the mammalian sterol-response element binding protein (SREBP) pathway to activate genes involved in sterol biosynthesis in response to low oxygen levels, while the other pathway involves the two-component hybrid histidine kinase protein Tco1. Mutant strains containing deletions of genes encoding components in either of these pathways were found to be less virulent in experimental mouse models. This study suggests that this pathogenic fungus experiences low levels of oxygen in the mammalian host, and that adaptation to these conditions is important for infection. Targeting components of the hypoxia response could yield more effective treatments for C. neoformans infections, which cause a large fraction of HIV/AIDS-related deaths worldwide. Notably, we find that mutants in the SREBP-like pathway are a hundred times more sensitive than wild-type cells to the widely used antifungal drug fluconazole.

## Introduction

Microbial pathogens must overcome numerous obstacles to infection to successfully colonize a host. It has been hypothesized that one of these barriers is hypoxia. Oxygen levels in mammalian tissues are found to be considerably below atmospheric levels [1–3]. Moreover, inflammation, thrombosis, and necrosis associated with infection are thought to lead to increased degrees of hypoxia [4,5]. While many pathogenic microbes are facultative or obligate anaerobes, a number of major bacterial and fungal pathogens are classified as obligate aerobes. Among these are the bacterial species *Neisseria meningiditis, Pseudomonas aeruginosa, Mycobacterium tuberculosis,* and Bordetella pertussis. In N. meningitidis and *B. pertussis,* mutants in homologs of the Escherichia coli hypoxia-responsive regulator Fnr are attenuated in experimental animals, consistent with a role for hypoxic adaptation in virulence [6,7]. Microarray analysis indicates that in *N. meningitidis,* Fnr activates genes encoding proteins involved in sugar transport/utilization, cytochromes, and denitrification enzymes that may promote usage of nitrate and nitrite instead of oxygen as a terminal electron acceptor during oxidative phosphorylation of ADP [6]. In contrast, a M. tuberculosis mutant defective in a nitrate reductase required for hypoxic growth in vitro was not attenuated in vivo [8].

In the fungal kingdom, some pathogens, such as *Candida albicans,* are capable of anaerobic fermentation in rich medium, but even in these species, molecular oxygen is thought to be essential for synthesis of ergosterol, NAD, and heme, molecules likely to be scarce in the host milleu and therefore difficult to obtain. In bacterial pathogens, NAD and heme synthesis can be catalyzed by alternative, oxygen-independent pathways, and sterol synthesis is thought to not exist [9,10]. Therefore, hypoxia should in principle be a far more significant challenge for fungal than bacterial pathogens; yet, it is unknown for any pathogenic fungi whether adaptation to hypoxia is necessary for virulence, and there is currently little information on the nature of corresponding adaptive mechanisms. In the best-studied human pathogenic fungus, *C. albicans,* one adaptive mechanism may involve the pleiotropic virulence regulator Efg1. Efg1 was recently shown in hypoxia to promote the synthesis of unsaturated fatty acids and to repress filamentous growth, whereas in normoxia, Efg1 promotes filamentation, activates glycolytic gene expression, and represses genes whose products promote oxidative metabolism [11,12].

Recent work in the nonpathogenic ascomycete fission yeast Schizosaccharomyces pombe has shown that a pathway homologous to the mammalian sterol-response element binding protein (SREBP) pathway is important for hypoxic adaptation [13,14]. In the mammalian pathway, sterols negatively regulate the release of a transcriptionally active, soluble DNA-binding portion of the SREBP transmembrane precursor protein through regulation of two proteolytic steps, one lumenal and one intramembrane [15]. Proteins required for cleavage of the mammalian SREBP precursor include SREBP cleavage-activating protein (SCAP), Insig, Site-1-protease, and Site-2-protease [15]. SCAP interacts with SREBP to regulate its cleavage and contains a domain thought to directly bind sterols, Insig negatively regulates SCAP, Site-1-protease is a member of the Kex2 family of lumenal proteases, and Site-2-protease is a metalloprotease responsible for intramembrane cleavage of the precursor [15]. The S. pombe SREBP-like pathway controls the expression of genes involved in ergosterol synthesis, and the available evidence suggests that hypoxia signals the SREBP-like pathway by reducing sterol levels, which, in turn, promotes the processing of the SREBP homolog, Sre1 [13,14].

One way of assessing whether a particular property of a pathogen is involved in virulence is to identify mutants defective in that property and then assess the pathogenicity of that mutant in an animal model of infection. For example, early work in C. albicans demonstrated that an engineered nonfilamentous mutant displayed greatly reduced virulence, providing initial evidence for a link between filamentous growth and virulence [16].

We have used the genetically tractable haploid human pathogenic yeast Cryptococcus neoformans to probe the role of hypoxic adaptation in fungal virulence. C. neoformans is an obligate aerobe that causes meningitis and other infections in immunocompromised individuals [17]. It exists as four distinct serotypes that are classified into three groups: *C. neoformans var grubii* (serotype A), *C. neoformans var gatii* (serotypes B and C), and *C. neoformans var neoformans* (serotype D). Together, these groups are estimated to cause between 15% and 40% of the 3.1 million annual deaths of individuals with HIV/AIDS; serotype A isolates are associated with the vast majority of infections in patients both positive and negative for HIV [18,19]. For the studies described below, we used the clinical serotype A isolate, H99.

## Results/Discussion

**Figure 1:**
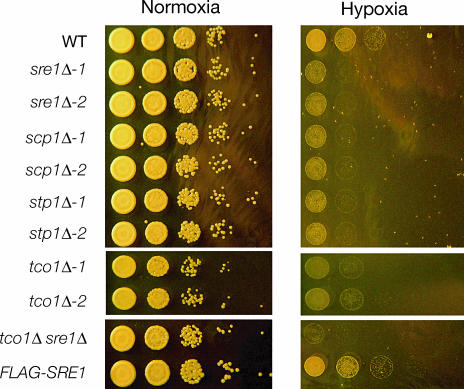
Mutants in SREBP Pathway and Tco1 Are Sensitive to Hypoxia Growth in normoxic and hypoxic conditions. Cultures diluted to OD_600nm_ = 0.6 were diluted serially in 10-fold increments prior to being spotted onto YPD plates. Plates were incubated in normoxic or hypoxic (controlled atmosphere chamber; less than 0.2% oxygen) conditions in the dark at 37 °C. Top and bottom panels are from the same plates, middle panels are from plates grown under the same conditions. WT, wild-type.

**Figure 2:**
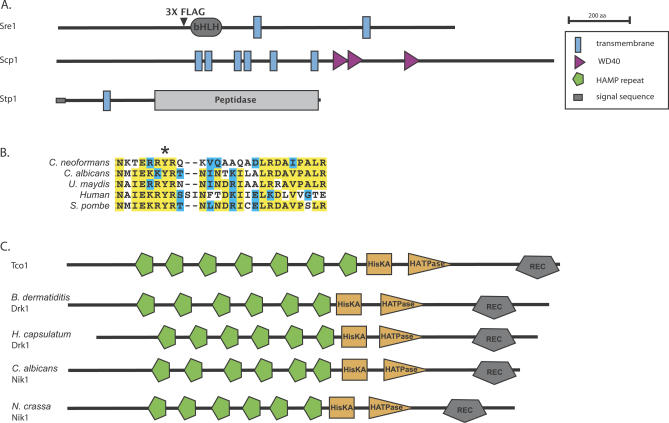
Predicted Protein Domains for SREBP Pathway Component Orthologs and Tco1 Histidine Kinase Family Members (A) Protein domains as predicted by SMART (http://smart.embl-heidelberg.de) for C. neoformans Sre1, Scp1, and Stp1. Transmembrane segments for Sre1 were predicted using MEMSAT3 (http://bioinf.cs.ucl.ac.uk). Arrowhead indicates site of 3×FLAG insertion into Sre1. bHLH, bHLH DNA-binding domain; peptidase, peptidase M50 family domain. (B) ClustalW sequence alignment between C. neoformans Sre1 and its orthologs in *C. albicans, Ustilago maydis, S. pombe,* and human (SREBP-1A), depicting a portion of the predicted DNA-binding domain. All five contain a conserved tyrosine residue (indicated by asterisk) specific to the SREBP family of bHLH transcription factors. (C) Domains as predicted by SMART for Tco1 homologs. C. neoformans Tco1, and orthologs to Tco1 from *B. dermatiditis, H. capsulatum, C. albicans,* and Neurospora crassa. HATPase, histidine kinase ATPase domain; HisKA, histidine kinase A domain; REC, cheY-like homologous receiver domain.

As part of an ongoing gene disruption project, we have generated targeted knockouts of ~1,200 C. neoformans genes (O. Liu, E. Chow, and H. Madhani, unpublished data). We screened this collection for mutants that displayed sensitivity to hypoxia, and we report here the characterization of four genes required for hypoxic adaptation. Among these are homologs of proteins that act in the mammalian SREBP pathway. We identified hypoxia-sensitive mutants in cryptococcal homologs of SREBP, Site-2-protease, and SCAP, which we respectively name Sre1, Stp1, and Scp1 (Figures 1 and 2A). Significantly, the basic helix-loop-helix (bHLH) DNA-binding domain of Sre1 contains a conserved tyrosine residue (Figure 2B) that is specific to the SREBP family of bHLH transcription factors [20]. We generated two independent knockouts of the genes encoding Sre1 *(sre1Δ-1* and *sre1Δ-2),* Stp1 *(stp1Δ-1* and *stp1Δ-2),* and Scp1 *(scp1Δ-1* and *scp1Δ-2),* and in each case, independent disruptions produced a hypoxia-sensitive phenotype (Figure 1). Re-introduction of *SRE1* into the *sre1Δ-2* strain restored sensitivity to levels similar to that of wild-type (Figure S1A).

As mentioned in the Introduction, it has been shown that in *S. pombe,* homologs of SREBP and SCAP are critical for survival under hypoxic conditions [13,14]. Our identification of a similar role for these components in the basidiomycete C. neoformans suggests broad conservation of this adaptive mechanism across the fungal kingdom. Curiously, examination of available databases yielded no clear homolog of Site-2-protease in the S. pombe genome sequence or in the genomes of fungi other than *C. neoformans,* suggesting that either this component of the SREBP pathway does not exist in those species or has diverged to a point where it is no longer recognizable. We were also unable to identify a homolog of Insig in *C. neoformans,* yet a clear homolog exists in S. pombe [14]. Finally, no ortholog of Site-1-protease is apparent in either genome; this function may be carried out by homologs of Kex2 present in each genome. Notably, the C. albicans protein most similar to Sre1 is Cph2 (Figure 2B), a transcriptional regulator previously shown to regulate hyphal development [21]. Although Cph2 binds to DNA elements similar to those bound by SREBP in mammalian cells [21], it is unclear whether it functions in an SREBP-like pathway.

In addition to components of the Sre1 pathway, we identified a hypoxia-sensitive mutant in Tco1, a member of a highly conserved family of fungal-specific histidine kinases (Figure 1). Tco1 has been shown previously to be required for virulence, yet the basis for this requirement has not been elucidated [22]. Paradoxically, Tco1 negatively regulates the expression of a known virulence factor, melanin formation, and, redundantly with Tco2, positively regulates the HOG MAPK pathway, which is dispensable for virulence [22]. We generated a second independent knockout of *TCO1,* which confirmed the hypoxia sensitivity of this mutant. Re-introduction of *TCO1* into the *tco1Δ-1* strain rescued its hypoxia-sensitive phenotype (Figure S1A). Significantly, a *tco1Δ sre1Δ* double knockout is more sensitive to hypoxia than either single mutant, indicating that Tco1 functions in a pathway parallel to the SREBP pathway (Figure 1).

Homologs of Tco1 with diverse functions have been described in a number of ascomycete fungi (Figure 2C). One of these, Drk1, has been recently shown in the human fungal pathogen Blastomyces dermatiditis to be required for temperature-regulated dimorphism and pathogenesis; its potential role in hypoxic adaptation was not reported [23]. A close homolog of Drk1 exists in Histoplasma capsulatum that is also involved in temperature-regulated dimorphism [23]. It was proposed that Drk1 is a transmembrane sensor that contains an N-terminal domain containing two transmembrane domains. However, analysis of Drk1 using the SMART database (Figure 2C) indicates that, instead, it contains a series of six tandem HAMP domains characteristic of the fungal-specific subfamily of histidine kinases, and is therefore more likely to be a soluble signaling protein. HAMP domains are found in a large number of signaling proteins, and, in one of these proteins, forms a four-helix bundle that may transduce signals via rotation of the helices [24]. In contrast to dimorphic fungi such as *B. dermatiditis, Coccidioides immitis,* and *H. capsulatum, C. neoformans* grows as a yeast both in the environment and in the host, suggesting a distinct role in virulence for Tco1 compared with that of its ascomycete orthologs.

**Figure 3:**
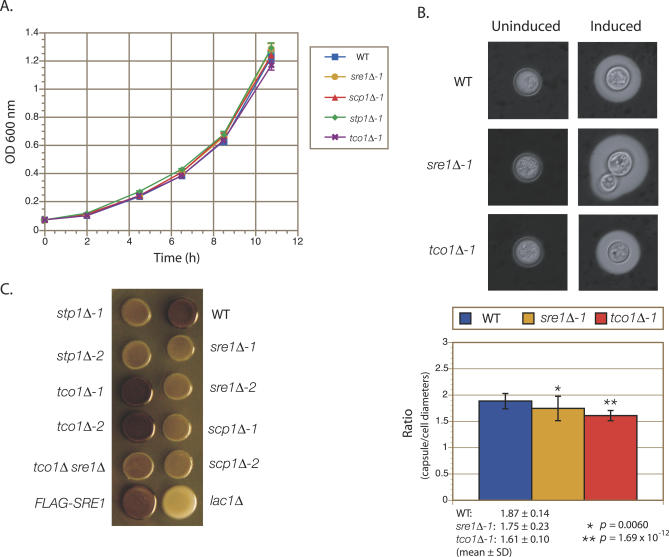
Phenotypic Characterization of Mutants in the SREBP Pathway and in *TCO1* (A) Growth in YNB medium at 37 °C. Growth was monitored via OD_600_ measurements. Data shown are an average of three independent cultures for each strain, and error bars represent standard deviations (SD). (B) Capsule assays. Above, capsule synthesis was induced in wild-type (WT), *sre1Δ-1,* and *tco1Δ-1* strains, and subsequently visualized using India ink staining. Images were taken at 160× magnification. Below, quantification of the ratio of capsule diameter to cell diameter for capsule-induced cells from the indicated strains. Data shown represent the mean ± SD, *n* ≥ 30. *p*-Values (asterisks) were derived using Student's *t*-test. (C) Melanin assays. The indicated strains were grown to saturation and spotted onto L-DOPA–containing medium. The plates were then cultured at 37 °C in the dark.

As with mutants in the Sre1 pathway, cells lacking Tco1 grow normally at 37 °C in minimal (yeast nitrogen base [YNB]) or rich (yeast extract peptone dextrose [YPD]) medium under normoxic conditions, suggesting that their defects in virulence are not due to a general growth defect (Figure 3A). We examined two characteristics believed to be important for virulence in *C. neoformans,* its polysaccharide capsule and its ability to melanize. We observed no gross defect in the formation of the polysaccharide capsule in any of the mutants; however, quantification of the ratios of capsule diameters to cell diameters did reveal rather subtle defects in the *sre1Δ* and *tco1Δ* mutants (Figure 3B). Mutants in the SREBP pathway display a slight defect in melanization under normoxic conditions and, as previously reported, mutants in *TCO1* display hypermelanization (Figure 3C). A *tco1Δ sre1Δ* double knockout also displays a slight defect in melanization. The complemented strains *sre1Δ-2 + SRE1* and *tco1Δ-1 + TCO1* displayed wild-type levels of melanization (Figure S1B).

**Figure 4:**
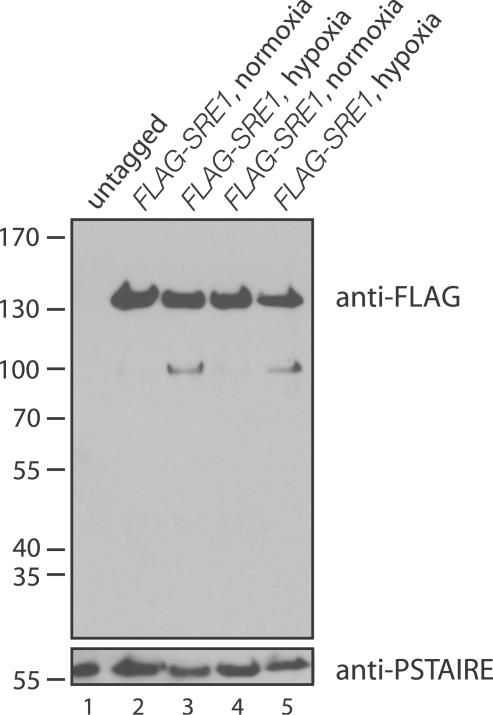
FLAG-Sre1 Is Processed in Low Oxygen Conditions Proteins extracts from wild-type and two independent cultures of *FLAG-SRE1* grown in normoxic and hypoxic (bubbling mixed gas into flasks; 0.2% oxygen) conditions were fractionated by SDS-PAGE and immunoblotted with antibodies against the FLAG epitope. Immunoblotting with antibodies against the cyclin-dependent protein kinase PSTAIRE was used to control for variation in loading.

In fission yeast, processing of Sre1 occurs in response to hypoxia [14]. To test whether this was the case in *C. neoformans,* we used homologous recombination to precisely insert three tandem copies of the FLAG epitope tag into the Sre1 coding sequence upstream of the predicted DNA-binding domain (Figure 2A). We assessed the processing of the protein by immunoblotting SDS-PAGE–fractionated extracts that were prepared from cells grown under normoxic versus hypoxic conditions (Figure 4). In response to hypoxia, a lower molecular weight form of Sre1 could be detected.

In mammalian cells, cleavage releases SREBP from endoplasmic reticulum and Golgi membranes, allowing it to then enter the nucleus and induce transcription of genes. It therefore seemed likely that hypoxia-dependent cleavage of Sre1 in C. neoformans would correlate with the up-regulation of hypoxia-regulated genes.

To test this hypothesis, we used whole-genome microarray-based transcriptional profiling. We found that that the response of wild-type cells to hypoxic conditions involved changes in the levels of 347 transcripts. These included the up-regulation of homologs of genes involved in stress and carbohydrate uptake/metabolism, similar to what has previously been reported in Saccharomyces cerevisiae [25], S. pombe [13], and C. albicans [11]. Select categories are presented in Table 1, and the full dataset is available in Table S3. In *S. cerevisiae, S. pombe,* and *C. albicans,* genes encoding respiratory proteins are down-regulated in response to hypoxia. In contrast, in *C. neoformans,* these genes are up-regulated, perhaps because it is an obligate aerobe. Similar to what has been described in other fungi, transcripts of genes involved in sterol, heme, and fatty acid metabolism are also up-regulated in C. neoformans in response to low oxygen conditions, including *SRE1* and homologs to the genes *ERG1, ERG3, ERG5, ERG25, HEM3, HEM13, SCS7, SUR2,* and *OLE1*. Categories of genes down-regulated in response to hypoxia include proteins involved in translation, vesicle trafficking, and cell wall and capsule synthesis *(CAP64* and *CAP60)*.

**Table 1:**
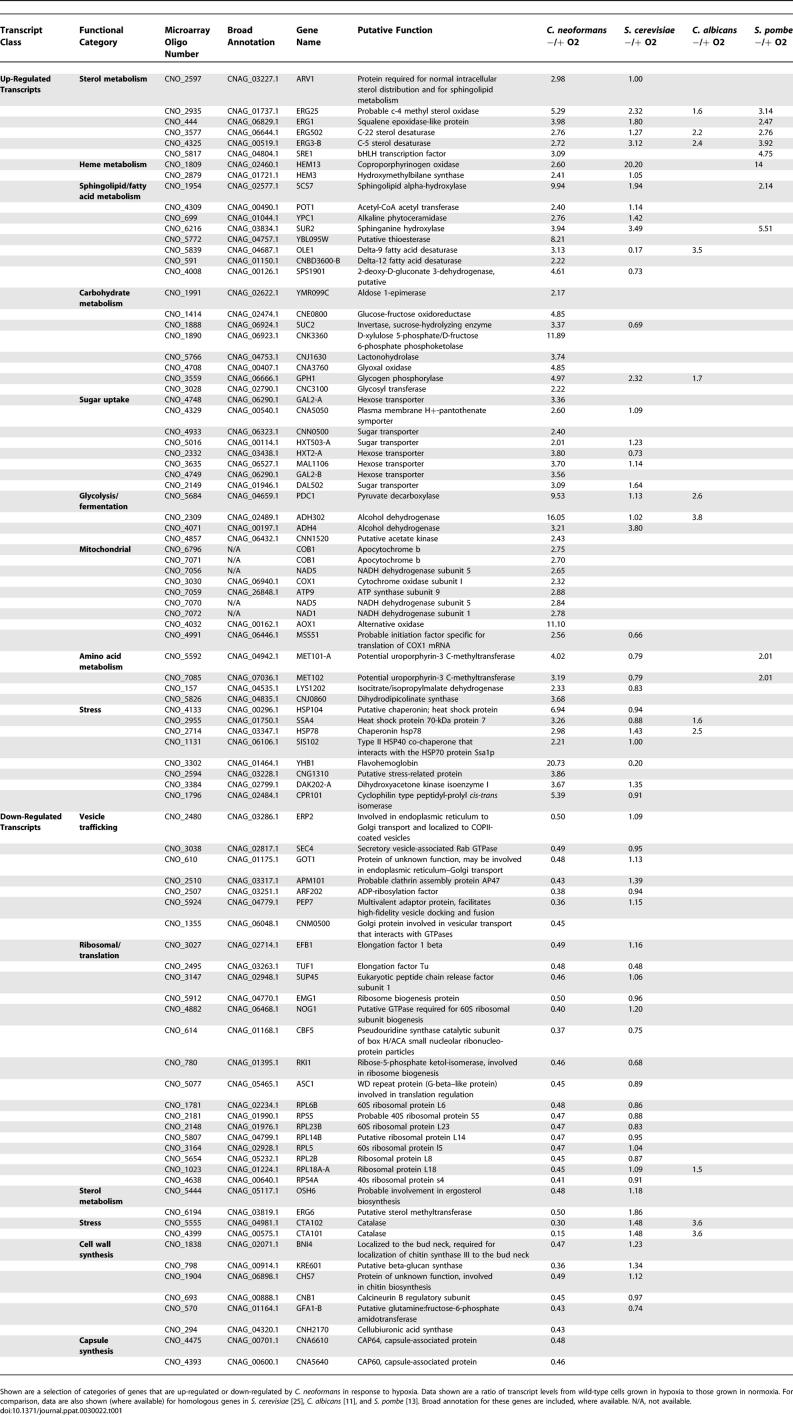
Select Categories of Genes Induced/Repressed by C. neoformans in Response to Hypoxia

**Figure 5:**
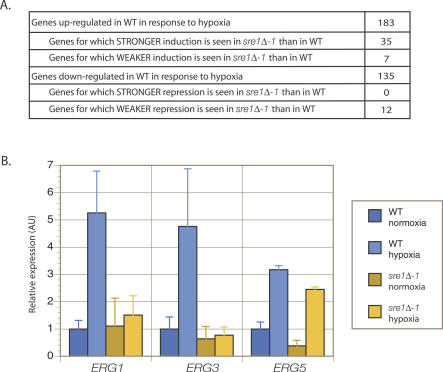
Microarray Hybridization and RT-QPCR Studies Demonstrate That a Subset of Genes Involved in the Hypoxia Response Require Sre1 for Their Regulation (A) Summary of profiling transcriptional response to hypoxia in *sre1Δ* and wild-type (WT) cells. cDNA from wild-type cells cultivated in normoxic conditions was hybridized against cDNA from wild-type cells exposed to hypoxic conditions on arrays containing 6,846 features from the genome of C. neoformans. Similarly, cDNA from *sre1Δ-1* grown in normoxia was hybridized against cDNA from *sre1Δ-1* grown in hypoxia. Statistical analysis of the resulting arrays was conducted using the software SAM. The transcriptional response to hypoxia in wild-type was identified as the set of genes with statistically significant changes in gene expression as determined by SAM. A cutoff of 2-fold or greater changes in gene expression was imposed upon this gene set to determine the number of genes up- and down-regulated in wild-type in response to hypoxia. The arrays hybridized with cDNA from *sre1Δ-1* were compared to those hybridized with wild-type cDNA to determine which hypoxia-induced transcriptional changes were significantly different between *sre1Δ-1* and wild-type. The resulting gene set is summarized above, where relative degree of induction/repression in *sre1Δ-1* and wild-type were compared by dividing fold-change in expression in *sre1Δ-1* with fold-change in expression in wild-type. (B) RT-QPCR. cDNA from *sre1Δ-1* and wild-type cells grown in normoxia and hypoxia were amplified using primers against *ERG1, ERG3, ERG5,* and *ACT1*. Values obtained for *ERG1, ERG3,* and *ERG5* were normalized against *ACT1* for each sample to give relative expression, and then expression for the three genes were normalized to their expression in wild-type in normoxic conditions. Error bars represent standard deviation across four samples.

A comparison in expression levels between wild-type and *sre1Δ-2* grown under normoxic conditions identified only two genes as having significant differences in expression between the two strains: *SRE1* itself and *ERG5,* a C-22 sterol desaturase involved in sterol synthesis (Table S5). While *ERG5* requires Sre1 for full expression under normoxic conditions, it does not require Sre1 for its induction under hypoxic treatment (Figure 5B).

We determined that of the 347 genes that were induced or repressed in wild-type by hypoxic treatment, 54 required Sre1 for their induction or repression (Table S4, summarized in Figure 5A). Of the subset denoting hypoxia-induced genes with weaker induction in *sre1Δ-1* than in wild-type, the greatest difference in expression between wild-type and mutant were in the two genes *ERG1* and *ERG3.* Strikingly, these genes encode for homologs to oxygen-dependent ergosterol biosynthetic pathway enzymes: *ERG1* encodes squalene epoxidase and *ERG3* encodes C-5 sterol desaturase. Both of these enzymes require molecular oxygen for their catalytic activities. We confirmed these data using reverse transcription quantitative PCR (RT-QPCR) analysis (Figure 5B).

We also performed similar experiments in strains lacking Tco1. In contrast to mutants in Sre1, the *tco1Δ* mutant did not show detectable defects in the regulation of mRNA levels for any of the 347 hypoxia-responsive genes (unpublished data). Thus, this kinase appears to function independently of transcription to mediate hypoxic adaptation, and we speculate that it may act post-transcriptionally. The phenotype of *tco1Δ* is specific in that strains containing disruptions of the other five histidine kinases encoded by the C. neoformans genome do not demonstrate sensitivity to hypoxia (C. Chun, unpublished observations).

Having identified mutants in two apparently distinct regulatory pathways required for hypoxic adaptation, we tested their effects on growth and virulence in infected experimental mice. To assess the effects on proliferation and survival in the host, we infected mice with 1:1 mixtures of wild-type and mutant cells and examined their relative representations in various tissues after a fixed period of time. Two infection routes were tested, intravenous and intranasal.

**Figure 6:**
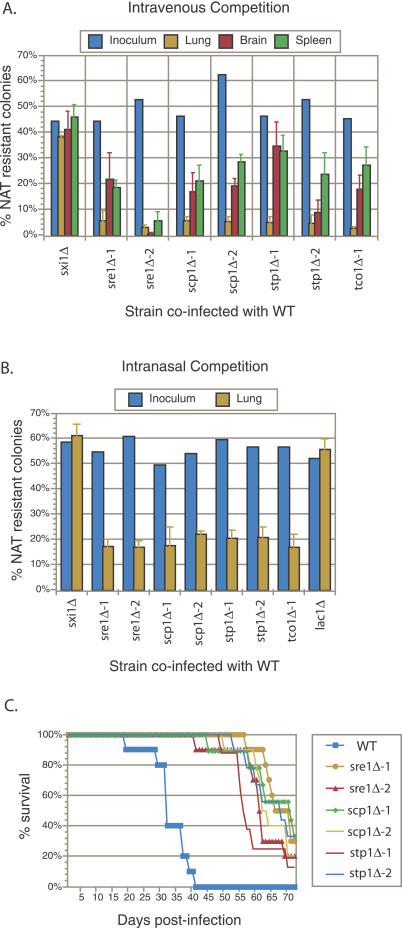
Hypoxia-Sensitive Mutants Display Proliferation and Virulence Defects (A) Tail vein inoculation competition experiments. Approximately 1:1 mixtures of wild-type (WT) and the indicated mutant (marked with a NAT resistance gene) were injected into mice (A/J) intravenously via the tail vein (2 × 10^6^ total cells/mouse). The actual proportion of mutant cells in each inoculum were determined by plating a dilution of the inoculum on nonselective medium and then assaying 100–200 individual colonies for NAT resistance. At 10 d post-infection, animals were sacrificed and the lungs, brains, and spleen from each animal were homogenized and serial dilutions were plated. Then, 100–200 colonies per organ were assayed for NAT resistance to determine the percentage of mutant cells. Error bars represent the standard deviation from four mice per inoculum. (B) Intranasal inoculation competition experiments. Approximately 1:1 mixtures of wild-type and the indicated mutant were inoculated intranasally into mice (A/J) (5 × 10^5^ total cells/mouse). The actual proportions of mutant cells were determined as in (A). At 21 d post-infection, animals were sacrificed and the lungs from each animal were treated as in (A). Error bars represent the standard deviation from four mice per inoculum. (C) Virulence assays. Eight to ten mice (A/J) were injected intravenously via the tail vein with 2 × 10^5^ cells of the indicated strain and progression to severe morbidity was monitored.

In the intravenous competition experiments, animals were sacrificed 10 d post-infection and the proportions of mutant cells in the lungs, brains, and spleens were determined. As shown in Figure 6A, knockout mutants in *SRE1, SCP1, STP1,* and *TCO1* showed a dramatic and reproducible defect in their ability to proliferate in lungs relative to wild-type cells. While they represented ~50% of the cells in the inoculum, the mutant cells made up only ~5% of the cells recovered from the lungs. A more moderate and variable defect was observed in the brains and spleens. As a control, we co-infected wild-type with a knockout mutant in *SXI1,* a gene involved in cell identity and sexual differentiation that is not expressed in non-mating cells and is dispensable for virulence [26,27]. In these control experiments, *sxi1*Δ represented ~50% of the cells in the inoculum and ~50% of the cells recovered from the various tissues.

In the intranasal competition experiments, animals were sacrificed 21 d post-infection and the proportion of mutant cells in the lungs was determined. Again, knockout mutants in *SRE1, SCP1, STP1,* and *TCO1* showed a significant and reproducible defect in their ability to proliferate in the lungs relative to wild-type cells (Figure 6B). A control infection using a 1:1 mixture of wild-type and *sxi1*Δ showed equal proliferation between wild-type and mutant. We also performed a co-infection using a mixture of wild-type and a knockout mutant in *LAC1,* which encodes for the laccase primarily responsible for melanin synthesis (Figure 6B). As previously reported, the *lac1Δ* mutant, which is completely defective in melanization (Figure 3C), displayed normal proliferation in the lungs [28].

A role for Tco1 in virulence was established previously using an intranasal inoculation assay [22]. To assess the role of SREBP pathway components in virulence, we inoculated eight to ten animals per strain by tail vein injection with two independently generated knockout mutants in *SRE1, SCP1,* and *STP1,* and monitored the mice for progression to severe morbidity. As shown in Figure 6C, each of the mutants displayed a similar and significant decrease in virulence. Thus, like the Tco1 pathway, the SREBP pathway plays a demonstrable role in pathogenesis.

**Figure 7:**
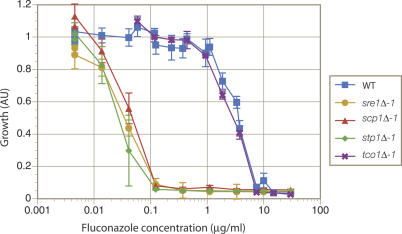
Mutants in the SREBP Pathway, but Not the *tco1Δ* Mutant, Display Hypersensitivity to Fluconazole Cells (10^4^ cells/ml) of wild-type and each mutant strain were cultured in YNB for three d in 96-well format in the presence of 0.0045–30 μg/ml fluconazole, after which relative growth was assessed using OD_600_ measurements. Data shown are an average of at least three independent cultures for each strain. Error bars represent standard deviations.

Azole antifungals are perhaps the most widely used agents for the treatment of fungal infections worldwide. They are thought to act solely as inhibitors of lanosterol-14α-demethylase, encoded in S. cerevisiae by *ERG11,* another oxygen-dependent enzyme in the ergosterol biosynthetic pathway. Our microarray analysis suggested a role for Sre1 in activating transcription of genes encoding several oxygen-dependent enzymes of this pathway, whereas Tco1 was dispensable. If these effects were relevant to growth, mutants in the Sre1 pathway should be sensitive to fluconazole treatment. Indeed, we found that mutation of *SRE1, STP1,* or *SCP1* results in a dramatic increase in sensitivity to fluconazole under normoxic conditions: ~100× lower concentrations of fluconazole were required to inhibit their growth compared with concentrations required for wild-type growth inhibition (Figure 7). In contrast, a knockout mutant of *TCO1* displayed a sensitivity that was identical to that of wild-type.

Fungal pathogens require molecular oxygen for the biosynthesis of several molecules, making hypoxic host tissues an inhospitable environment. Using the obligate aerobic human pathogen C. neoformans as a model, we have identified two pathways that are required for hypoxic adaptation and virulence. We show that an SREBP-like pathway in C. neoformans is activated by hypoxia, is required for hypoxic adaptation, and controls the expression of oxygen-dependent enzymes in the ergosterol biosynthetic pathway. The Sre1 pathway acts in parallel with a pathway controlled by the histidine kinase homolog Tco1 in hypoxic adaptation. Importantly, analysis of mutants clearly demonstrates both are required for pathogen growth/survival in experimental animals and for virulence, which strongly suggests a link between hypoxic adaptation and pathogenesis. The simplest interpretation of our data is that the pathogen experiences hypoxia in the host to which it must adapt in order to cause disease.

Other pathways are also likely to play a role in hypoxic adaptation. For example, transcription of *OLE1,* which encodes for a Δ9-fatty acid desaturase in *S. cerevisiae,* is regulated in a manner that shows remarkable parallels to the Sre1 pathway. In response to low levels of oxygen or unsaturated fatty acids, a proteolytic cleavage event releases the putative transcription factor Mga2 from the endoplasmic reticulum membrane, allowing up-regulation of *OLE1* transcription [29]. Indeed, our microarray data shows that *OLE1* is up-regulated in C. neoformans in response to hypoxia in an Sre1-independent manner.

Although our results show that hypoxia-sensitive mutants in two distinct pathways are defective in virulence, it is conceivable that the dual requirement for the Sre1 and Tco1 pathways in virulence reflects defects other than hypoxic adaptation in the corresponding mutants. However, our data argue that the requirement for these pathways cannot be explained by a trivial defect for either pathway for growth in minimal medium at 37 °C (Figure 3A). Furthermore, the mutants do not display gross defects in capsule formation, and they display phenotypes during murine infection distinct from those defective in the production of two other virulence factors, melanin and urease [28,30]. Specifically, while SREBP pathway mutants display a slight defect in melanin formation, their reduced growth in the lungs of mice infected intranasally contrasts to that of *lac1*Δ mutants, which are completely defective in melanization yet display normal proliferation in the lungs both in published work [28] and in our hands (Figure 6B). Likewise, mutants defective in urease activity have been reported to not display a defect in proliferation in the lungs [30], in contrast to the effects of *sre1*Δ and *tco1*Δ mutants reported here. While distinct from mutants defective in melanin and urease production, the tissue proliferation/survival profiles of SREBP and Tco1 pathway mutants are similar to each other (Figure 6), consistent with a common mechanism underlying their virulence defects.

For each mutant strain tested, we observed a greater fold-defect in the lungs compared with that in the brain or spleen. At least two explanations could account for these observations. First, the hypoxic conditions within the lungs could be more severe than in the brain or spleen, perhaps due to the fact that the lungs are a predominant site of inflammation. The influx of large numbers of activated macrophages into the lungs may generate a locally hypoxic microenvironment that is more challenging than those of the brain or spleen. Alternatively, turnover rate of C. neoformans may be greater in the lungs than in the brain and spleen, which would amplify any per-division growth differences between wild-type and mutant strains. Assuming similar steady-state levels of organisms, higher turnover would require both a higher growth rate of C. neoformans in the lungs as well as a greater degree of killing than in the non-lung tissues. Further studies will be required to distinguish between these possibilities.

The remarkable sensitivity of Sre1 pathway mutants to fluconazole is relevant for two reasons. First, it suggests that the effectiveness of antifungals that target ergosterol biosynthesis may in part be aided in vivo by the sensitivity of ergosterol synthesis to hypoxic conditions. This model may explain observations in otherwise puzzling reports showing that while fluconazole blood levels typically only reach 10–20 μg/ml, successful treatment of C. albicans isolates that display in vitro resistance towards fluconazole (MIC > 50 μg/ml) can be achieved in a substantial fraction of cases [31]. It may be that hypoxic conditions in vivo lower the effective concentration of the drug required to clear the infection. Second, our data suggest that if inhibitors of the Sre1 pathway could be developed, they would strongly synergize with ergosterol pathway inhibitors such as azoles in the treatment of cryptococcal infections.

## Materials and Methods

### Strains and media.

C. neoformans serotype A strain H99 (a kind gift of J. K. Lodge) was used as wild-type. Strains were grown in rich medium, YPD (1% yeast extract, 2% Bacto-peptone, 2% glucose, 0.015% L-tryptophan, 0.004% adenine) or minimal medium, YNB (0.45% yeast nitrogen base w/o amino acids w/o ammonium sulfate, 1.5% ammonium sulfate, 2% glucose). The strains used in this study are listed in Table S1.

### Strain engineering.

The wild-type H99 strain was transformed using biolistic techniques as previously described [32]. The gene-specific deletion constructs were generated with the primers listed in Table S2 using overlap fusion PCR. Specifically, the nourseothricin (NAT) resistance *(natR)* cassette was amplified from plasmids derived from pHL001 using the primers 5′NAT-10 and 3′NAT-10. This cassette was targeted to delete specific genes by fusing the cassette to the genomic sequences flanking the target gene. These flanking sequences were amplified from H99 genomic DNA by PCR using the ***-W1 and ***-W3 primers to amplify a ~1-kb region 5′ to the target gene and the ***-W4 and ***-W6 primers to amplify a ~1-kb region 3′ to the target gene. The two flanking regions and the *natR* cassette were fused into one product using PCR and biolistically transformed. Transformants were selected on YPD agar plates containing 100 μg/ml NAT. *sre1*Δ*-1* was generated using the CN4363 set of primers and deletes a portion of *SRE1,* including the DNA-binding domain. *sre1*Δ*-2* was generated using the CDS_5817 set of primers and deletes all of *SRE1*. *scp1*Δ*-1* was generated using the CN1329 set of primers and deletes a portion of *SCP1* that includes transmembrane domains. *scp1*Δ*-2* was generated using the CDS_2788 set of primers and deletes all of *SCP1*. *stp1*Δ*-1* and *stp1*Δ*-2* were both generated using the CDS_3547 set of primers, which deletes all of *STP1*. *stp1*Δ*-1* and *stp1*Δ*-2* were independently generated in separate biolistic transformations. *tco1*Δ*-1* was generated using the CN1538 set of primers and deletes all but ~200 bp at the 5′ end of the open reading frame (ORF). *tco1*Δ*-2* was generated using the CDS_3015 set of primers and deletes all of *TCO1*. *sxi1*Δ was generated using the CN5693 set of primers and *lac1*Δ was generated using the CN2897 set of primers. Both of these deletions result in a complete deletion of the gene. Strains referred to as complete deletions may leave intact small portions of the 5′ or 3′ ends of the ORF due to ambiguities in the annotation, but are not likely to retain any function. The recombination events were verified by PCR using the ***-V5 and VER-5-3 primers to detect the 5′ integration event and the ***-V3 and VER-3-2 primers to detect the 3′ integration event. The *tco1*Δ*sre1*Δ double mutant was generated by using a neomycin (NEO) resistance *(neoR)* cassette which was amplified from the plasmid pJAF1 using the primers 5′NAT-10 and 3′NEO-10. The *neoR* cassette was fused to flanks generated using the CDS_5817 set of primers. This knockout construct was then transformed into the *tco1*Δ*-1* strain. Transformants were selected on YPD agar plates containing 200 μg/ml G418.

As a redundant test to confirm the phenotypes of independent disruptants, the *sre1*Δ*-2* and *tco1*Δ*-1* mutants were complemented by biolistically transforming the knockout strains with two linear DNA constructs simultaneously. The first construct restored *SRE1* or *TCO1* to its original locus, replacing the *natR* cassette which had been used to disrupt the gene. The second construct inserted a *neoR* cassette ~300 bp upstream of the start of the *SRE1* ORF, or ~1,100 bp upstream of the start of the *TCO1* ORF. Specifically, in order to complement *sre1*Δ*-2,* the primers CDS_5817-W1 and CDS_5817-W6 were used to amplify the *SRE1* ORF from H99 genomic DNA, including 1 kb of upstream and downstream sequence, creating the first transformation construct. The second transformation construct was generated using overlap fusion PCR. The primers C_p5817-W1h and C_p5817-W3 were used to generate the ~1-kb region downstream of the *neoR* insertion site, and the primers C_CPR202-W4 and C_CPR202-3F were used to amplify the ~1-kb region upstream of the *neoR* insertion site. These two flanking regions were fused to the *neoR* cassette, which was amplified as above, creating the second transformation construct. In order to complement *tco1*Δ*-1,* the primers CDS_3015-W1 and CDS_3015-W6 were used to amplify the *TCO1* ORF from H99 genomic DNA, including 1 kb of upstream and downstream sequence, creating the first transformation construct. The second transformation construct was generated using overlap fusion PCR. The primers C_p3015-W1 and C_p3015-W3 were used to generate the ~1-kb region downstream of the *neoR* insertion site, and the primers C_p3015-W4 and C_p3015-W6 were used to amplify the ~1-kb region upstream of the *neoR* insertion site. These two flanking regions were fused to the *neoR* cassette, creating the second transformation construct. *sre1*Δ*-2* and *tco1*Δ*-1* were biolistically transformed with both constructs simultaneously, and transformants were selected on YPD agar plates containing 200 μg/ml G418. Positive transformants were patched onto YPD agar plates containing 200 μg/ml G418 and then replica-plated onto YPD agar plates containing 100 μg/ml NAT. Colonies that were resistant to G418 and sensitive to NAT were screened using PCR for the presence of the complete *SRE1* or *TCO1* ORF. The phenotypes of complemented strains are shown in Figure S1.

### Generation of the 3×FLAG-tagged *SRE1* strain.

Three copies of the FLAG epitope plus linker sequence (REQKLEL-3×FLAG-GSGSGS) were inserted between A313 and A314 of Sre1 just upstream of the predicted DNA-binding domain. Insertion of the 3×FLAG construct was performed by biolistically transforming H99 with two linear DNA constructs simultaneously. The first construct targeted the *natR* cassette for insertion ~300 bp upstream of the start of the *SRE1* ORF, just downstream of the adjacent gene. The second construct targeted the 3×FLAG sequence for insertion into the *SRE1* ORF but did not contain any resistance marker. Both constructs were generated using overlap fusion PCR. Specifically, the *natR* cassette was amplified as described above. The primers C_5817-W1FLg and C_5817-W3 were used to amplify the ~1,300-bp region between the site of insertion of the *natR* cassette and the site of insertion for the 3×FLAG sequence. The primers C_CPR202-W4 and C_CPR202-3F were used to amplify the ~1-kb region upstream of the *natR* insertion site. These two flanking regions were fused to the *natR* cassette using overlap PCR to create one of the two transformation constructs. The 3×FLAG sequence was amplified by PCR from the p3FLAG-KanMX [33] plasmid using the primers C_5817-FLAGFg and C_5817-FLAGRg. C_5817-W1Flg and C_5817-W3 were again used to amplify the upstream flanking region between the 3×FLAG insertion site and the *natR* insertion site, and the primers C_5817–5FgFl and C_5817–5Re were used to amplify the ~1-kb region downstream of the 3×FLAG insertion site. These two flanks were fused to the 3×FLAG cassette using overlap PCR to create the second transformation construct. After biolistic transformation with both constructs simultaneously and selection for NAT resistance, transformants were screened for insertion of the 3×FLAG sequence by PCR using the primers C_5817V5Fle and C_5817-FLAGFg.

### Hypoxic cultivation.

Strains were grown in YPD at 37 °C. Normoxic conditions were considered general atmospheric levels within the lab (~21% O_2_). For hypoxic conditions, two systems were employed. One system consisted of a controlled atmosphere chamber (855-AC; Plas-Labs, http://www.plas-labs.com) maintained at 37 °C and kept at below 0.2% oxygen levels utilizing a gas mixture containing 5% CO_2_, 10% H_2_, 85% N_2_ in combination with palladium catalyst. While the hydrogen–palladium mixture eliminated most oxygen within the chamber, the architecture of the chamber did allow a small amount of oxygen to slowly enter the chamber, allowing for C. neoformans cultivation (as it is an obligate aerobe). Oxygen levels within the controlled atmosphere chamber were monitored using a QRAE Plus oxygen detector (RAE Systems, http://www.raesystems.com). Low oxygen (and not high CO_2_) was the most likely source of the phenotypes presented in this paper, as all strains grew normally in atmospheric conditions that were supplemented with 5% CO_2_ (Figure S2). The second system utilized a gas mixture containing 0.2% O_2_, 0.25% CO_2_, 99.55% N_2_ that was bubbled through sterile pipettes into medium contained in silicon-stoppered flasks arranged in parallel. The gas mixture was humidified prior to being bubbled into the medium by first bubbling through sterile water to minimize medium loss.

For growth plate phenotypes, cultures grown in YPD at 37 °C were diluted to OD_600 nm_ = 0.6 in water, then diluted 10-fold in series prior to being spotted onto YPD agar plates. Plates were then cultured in the dark in normoxic or in the controlled atmosphere chamber in hypoxic conditions.

For the growth curve, cultures were grown overnight in liquid YNB medium and diluted to OD_600 nm_ = 0.085 in YNB. These were then cultured at 37 °C in a roller drum. At the indicated time intervals, cell density was assessed via OD_600_ readings. Data shown are an average of three independent experiments and error bars represent their standard deviations.

### Capsule assay.

C. neoformans strains were grown in liquid YPD cultures overnight at 30 °C. The cultures were then diluted 1/100 in either Sabouraud medium (non-inducing conditions) or 10% Sabouraud medium buffered to pH 7.3 with 50 mM MOPS (capsule-inducing conditions) and grown at 30 °C for 3 d. An equal volume of culture and India ink were mixed and the capsule was visualized using bright-field microscopy at 160× magnification using an Axiovert 200M (Zeiss, http://www.zeiss.com) microscope running Axiovision software. To quantitate capsule size, the cell diameter and capsule diameter of at least 30 cells per strain under capsule-inducing conditions were measured using the Axiovision software. Statistical analysis was by Student's *t-*test.

### Melanization assays.

Cultures grown overnight in YPD medium were spotted onto melanin-inducing plates containing L-DOPA (L-dihydroxyphenylalanine, 100 mg/l; Sigma, http://www.sigmaaldrich.com) and grown for 3–5 d in the dark at 37 °C.

### Isolation of total RNA.

Log-phase cultures were grown aerobically in YPD at 37 °C, with shaking, to an OD_600 nm_ = 0.6, at which point the cultures were split into two. For hypoxic growth, cells were collected by centrifugation and then resuspended while in the controlled atmosphere chamber with 50 ml of YPD medium that had been previously deoxygenated by >2 h incubation with agitation in chamber. For normoxic growth, cultures were maintained in atmospheric conditions. Following 2 h of hypoxic treatment, both normoxic and hypoxic cultures were collected by centrifugation at 4 °C. Cell pellets were flash frozen in liquid nitrogen and lyophilized prior to total RNA extraction using TRIzol Reagent (Invitrogen, http://www.invitrogen.com) following manufacturer's instructions. RNA samples were treated with DNase I (Roche, http://www.roche.com) for 30 min at 37 °C, followed by two extractions with chloroform, precipitation in isopropanol, and resuspension in DEPC-treated water.

### Microarray-based genome-wide transcriptional profiling.

Total RNA was reverse transcribed by priming with oligo dT and utilizing aminoallyl-dUTP. The resultant cDNA was then coupled to Cy3- and Cy5-labeled probes (Amersham Biosciences, http://www.amershambiosciences.com), and hybridized to microarrays printed on site, containing 6,846 70-mer oligonucleotides corresponding to C. neoformans ORFs from the serotype A genome as annotated by our laboratory (http://cryptogenome.ucsf.edu). Labeled cDNA from wild-type grown in normoxic conditions was hybridized against cDNA from wild-type grown in hypoxic conditions; cDNA from *sre1Δ-1* grown in normoxia was hybridized against *sre1Δ-1* grown in hypoxia; cDNA from *tco1Δ-1* grown in normoxia was hybridized against *tco1Δ-1* grown in hypoxia; and cDNA from *sre1Δ-2* grown in normoxia was hybridized against wild-type grown in normoxia. Data for each strain represents four independent experiments and includes two dye-swaps. Arrays were scanned on an Axon 400B scanner with GenePix software (Axon Instruments, http://www.moleculardevices.com) and the images generated were analyzed using SpotReader software (Niles Scientific, http://www.nilesscientific.com) for spot identification and flagging. Array signals were bulk-normalized and filtered for flagged spots using NOMAD (available at http://ucsf-nomad.sourceforge.net). Data were log-transformed (base 2) and filtered for genes that contained data for at least three out of four arrays from each strain, and missing values were calculated through K-nearest neighbor algorithm using Significance Analysis of Microarrays (SAM) software [34] prior to statistical analysis by SAM. The class of genes up- and down-regulated in wild-type was determined in a one-class response test based on the arrays hybridized with cDNA from wild-type. Genes were defined as being significantly regulated in wild-type if they were scored by SAM as significant with a median false discovery rate (FDR) of less than 8% and if their expression changed by at least 2-fold or greater. For comparison of the differences in transcriptional response to hypoxia in wild-type and mutant, a two-class response test was employed, comparing the arrays hybridized with wild-type cDNA to either the *sre1Δ-1* arrays or the *tco1Δ-1* arrays. All SAM analysis employed an FDR of less than 8%. The comparison between wild-type and *tco1Δ-1* arrays at this FDR did not return any genes with statistically significant differences between the two strains. The data from the set of genes returned from the comparison between wild-type and *sre1Δ-1* were averaged across the four arrays per strain, and the average fold-change in expression in *sre1Δ-1* was divided by the average fold-change in expression in wild-type for each gene. For the set of genes up-regulated by wild-type in response to hypoxia, if this value was greater than 1, the gene was defined as having stronger induction in *sre1Δ-*1 than in wild-type, whereas if the value was less than 1, the gene was defined as having weaker induction in *sre1Δ-1* than in wild-type. For the set of genes down-regulated by wild-type in response to hypoxia, if this value was greater than 1, the gene was defined as having weaker repression in *sre1Δ-*1 than in wild-type, and if the value was less than 1, the gene was defined as having stronger repression in *sre1Δ-*1 than in wild-type. Statistically significant genes identified by SAM with 2-fold or greater changes in expression are listed in Tables S3 and S4 in Supporting Information.

### Gene naming conventions.

We refer to the generic names described by the Stanford B-3501 serotype D genome annotation. These are in the form CNXY, where X indicates the chromosome (A for Chromosome I, B for Chromosome II, etc.) and Y indicates the gene position. In addition, if a gene has a homolog in S. cerevisiae with a BLASTP expect value of less than 10^−4^, we refer to it by the name of the best S. cerevisiae homolog. In cases where the same gene in S. cerevisiae was the closest homolog to multiple genes in *C. neoformans,* two digits were added to the end of the S. cerevisiae name to generate unique specific names (e.g., *SPS1901* is one C. neoformans gene homologous to the S. cerevisiae gene *SPS19*) (E.Chow, O. Liu, and S. O'Brien, unpublished data).

### RT-QPCR.

Microarray results were confirmed utilizing RT-QPCR. cDNA was synthesized from the same DNase I–treated total RNA that was used for microarray analysis using Superscript III Reverse Transcriptase (Invitrogen) and oligo dT primers. Approximately 0.2 ng of cDNA was used as template in a QPCR reaction containing SYBR Green dye (Molecular Probes, http://probes.invitrogen.com). Fluorescent signal was measured on an Opticon DNA Engine PCR machine (MJ Research, http://www.bio-rad.com). For each primer set, standard curves were generated using 5-fold sequential dilutions of cDNA to account for differences in priming efficiencies. For each sample, values obtained were normalized to the levels of actin *(ACT1)*. Data shown are averages of the four independent samples, and are normalized to the levels of transcript present on average in wild-type under normoxic conditions. The following primers were used: for *ERG1,* C_ERG1-3-5 and C_ERG1-5-5; for *ERG3,* C_ERG3-3-5 and C_ERG3-5-5; for *ERG5,* C_ERG5-3-1 and C_ERG5-5-1; for *ACT1,* C_ACT1–1 and C_ACT1–2.

### Immunoblotting.

Strains containing *FLAG-SRE1* as the sole allele were grown to OD_600 nm_ = 0.4 in 100 ml YPD at 37 °C. For the normoxic growth sample, half the culture was then harvested. For hypoxic growth, the remaining culture was spun down and the cell pellet was then resuspended in 50 ml YPD medium that had been previously de-oxgenated for >2 h by the bubbling of gas (a mixture containing 0.2% O_2_, 0.25% CO_2_, 99.55% N_2_) through sterile pipettes into the medium contained in silicon stopper-sealed flasks. The cultures were then cultivated in the presence of bubbling gas mixture for an additional 2 h before harvesting. Both normoxic and hypoxic cultures were pelleted by centrifugation, and following a wash with cold water, were flash frozen in liquid nitrogen and lyophilized. The lyophilized cells were lysed in 24 mM NaOH and 1% (v/v) β-mercaptoethanol on ice for 30 min, and proteins were precipitated with 6.4% trichloracetic acid while on ice. After centrifugation, the protein pellet was washed once with acetone, dried, and then resuspended in HU buffer (200 mM phosphate buffer [pH 6.8], 8M urea, 5% SDS, 1 mM EDTA, 100 mM DTT, bromophenol blue) and loaded onto a bis-Tris gel. Following SDS-PAGE, the fractionated proteins were transferred to nitrocellulose membrane (MSI Nitrobind membrane; Fisher Scientific, https://www1.fishersci.com). Immunoblotting was performed using mouse anti-FLAG IgG (Sigma) and rabbit anti-PSTAIRE IgG (Santa Cruz Biotechnology, http://www.scbt.com), which were recognized by horseradish peroxidase-conjugated goat anti-mouse IgG and goat anti-rabbit IgG secondary antibodies (Bio-Rad, http://www.bio-rad.com), respectively. Visualization was performed utilizing SuperSignal West Pico Chemiluminescent Substrate (Pierce, http://www.piercenet.com).

### Intravenous co-infection experiments.

C. neoformans strains were individually grown in liquid YPD cultures overnight at 30 °C. Cells were counted using a hemacytometer and an equal number of wild-type cells and cells of the co-infecting strain were combined. The cells were washed twice in PBS and resuspended in PBS to a final concentration of 2 × 10^7^ cells/ml. Then, 5–6 wk-old female A/J (National Cancer Institute [NCI]) mice were anesthetized with inhaled isoflurane, and 100 μl of the inoculum (2 × 10^6^ total cells) was injected via tail vein. Four mice were infected per inoculum. A dilution of each inoculum was also plated on Sabouraud agar plates containing 40 μg/ml gentamicin and 50 μg/ml carbenicillin. The plates were incubated for 2 d at 30 °C, colonies were counted to verify the concentration of cells in the inoculum, and the proportion of mutant cells in the inoculum was determined by assaying 100–200 colonies for NAT resistance on YPD agar plates containing 100 μg/ml NAT. Mice were sacrificed by CO_2_ inhalation followed by cervical dislocation 10 d post-infection, and the lung, brain, and spleen were removed and homogenized in 5 ml sterile PBS. Serial dilutions of each organ sample were plated on Sabouraud agar plates containing 40 μg/ml gentamicin and 50 μg/ml carbenicillin. The plates were incubated for 2 d at 30 °C, colonies were isolated, and the proportion of mutant cells within each organ was determined by assaying 100–200 colonies for NAT resistance on YPD agar plates containing 100 μg/ml NAT.

### Intranasal co-infection experiments.

Inocula were prepared as in the intranvenous co-infection experiments except cells were resuspended in PBS to a final concentration of 1 × 10^7^ cells/ml. Then, 4–5 wk-old female A/J (NCI) mice were anesthetized by intraperitoneal injection of ketamine (75 mg/kg) and medetomidine (0.5–1.0 mg/kg). The mice were then suspended from a silk thread by their front incisors and 50 μl of the inoculum (5 × 10^5^ cells) were slowly pipetted into the nares. After 10 min, the mice were lowered and the anesthesia was reversed by intraperitoneal injection of atiplamezole (1.0–2.5 mg/kg). Four mice were infected per inoculum. The concentrations of the inocula and the proportions of mutant cells in the inocula were determined as in the intravenous co-infection experiments. After 21 d post-infection, the animals were sacrificed by CO_2_ inhalation followed by cervical dislocation and the lungs were analyzed as in the intravenous co-infection experiments.

### Mouse virulence studies.

Inocula were prepared as in the intravenous-co-infection experiments, except cells were resuspended in PBS to a final concentration of 2 × 10^6^ cells/ml. Then, 5–6 week-old female A/J (NCI) mice were anesthetized with inhaled isoflurane and 100 μl of the inoculum (2 × 10^5^ cells) were injected via the tail vein. The concentration of cells in the inoculum was confirmed by plating serial dilutions. Mice were monitored several times a week until onset of symptoms (weight loss, ruffled fur, abnormal gait) and then monitored daily. Mice that displayed signs of severe morbidity (weight loss, abnormal gait, hunched posture, swelling of the cranium) were sacrificed by CO_2_ inhalation followed by cervical dislocation.

### Fluconazole sensitivity assay.

Strains were cultured overnight in YNB medium at 30 °C, spun down, washed once in PBS, and then resuspended in YNB to a concentration of 10^4^ cells/ml. This resuspension was aliquoted into 96–deep well plates at a volume of 500 μl/well. To this, 100 μl of fluconazole (Sigma; stock solution of 1 mg/ml prepared in dimethyl sulfoxide [DMSO] and subsequently diluted in YNB) was added to give final fluconazole concentrations ranging from 0.0046 to 30 μg/ml. The plates were cultured for 48 h at 30 °C with shaking (1,000 rpm), at which point measurements were taken on a spectrophotometer. For each culture, 100 μl of YNB was added to one well instead of fluconazole. The OD_600_ reading for this well was set to a value of one, and growth in all other wells for that culture were normalized to that reading. For each strain, appropriate dilutions of DMSO without fluconazole were added to a series of cultures as a negative control. Growth with DMSO for all strains at all concentrations tested was similar to growth without DMSO (unpublished data).

## Supporting Information

Figure S1 Complementation of Hypoxia-Sensitivity and Melanization Phenotypes *sre1Δ* and *tco1Δ* Strains by Homologous Targeting of the Corresponding Wild-Type Genes (A) *SRE1* was re-introduced into its endogenous locus in *sre1Δ-2* and *TCO1* was re-introduced into its endogenous locus in *tco1Δ-1*. Cultures diluted to OD_600nm_ = 0.6 were diluted serially in 10-fold increments prior to being spotted onto YPD plates. Plates were incubated in normoxic or hypoxic (controlled atmosphere chamber; less than 0.2% oxygen) conditions in the dark at 37 °C.
(B) Melanin assays. The indicated strains were grown to saturation and spotted onto L-DOPA–containing medium. The plates were then cultured at 37 °C in the dark.
(4.1 MB PDF)

Figure S2 Knockout Mutants in SREBP Pathway Components and in *TCO1* Are Not Sensitive to High Levels of Carbon Dioxide Cultures diluted to OD_600nm_ = 0.6 were diluted serially in 10-fold increments prior to being spotted onto YPD plates. Plates were incubated in the dark at 37 °C in normal atmospheric conditions or in air supplemented with 5% CO_2_ (NuAire IR Autoflow CO_2_ Water-Jacketed Incubator, http://www.nuaire.com).
(7.9 MB PDF)

Table S1 Strains Used in This Study (38 KB DOC)

Table S2 Primers Used in This Study (114 KB DOC)

Table S3 Microarray Dataset (103 KB XLS)

Table S4 Sre1-Regulated Genes in Hypoxia (35 KB XLS)

Table S5 Sre1-Regulated Genes in Normoxia (15 KB XLS)

### Accession Numbers

Microarray data described in this paper has been submitted to the National Center for Biotechnology Information (NCBI) Gene Expression Omnibus database (http://www.ncbi.nlm.nih.gov/geo) under accession number GSE6226. The GenBank (http://www.ncbi.nlm.nih.gov/Genbank/index.html) accession number for the sequences discussed in this paper are as follows: Stanford B-3501 serotype D genome (AAEY00000000); Sre1 homologs from C. albicans (AAK69672), human (SREBP-1A, NP_001005291), S. pombe (CAB52036), and *Ustilago maydis* (XP_761868); and Tco1 homologs from B. dermatiditis (ABF13477), *C. albicans* (BAA24952), and *Neurospora crassa* (AAB03698). Where GenBank accession numbers were not available, we have provided annotation by the Broad Institute of MIT and Harvard (http://www.broad.mit.edu): C. neoformans Scp1 (Broad locus CNAG_01580.1), Sre1 (Broad locus CNAG_04804.1), Stp1 (Broad locus CNAG_05742.1), and Tco1 (Broad locus CNAG_01850.1); and H. capsulatum ortholog to Tco1 (Broad locus HCAG_4501.1).

## Abbreviations

bHLH: basic helix-loop-helix
NAT: nourseothricin
NEO: neomycin
ORF: open reading frame
RT-QPCR: reverse transcription quantitative PCR
SAM: Significance Analysis of Microarrays
SCAP: SREBP cleavage-activating protein
SREBP: sterol-response element binding protein
YNB: yeast nitrogen base
YPD: yeast extract peptone dextrose
