# Supplementary figures and images for: A Link between Virulence and Homeostatic Responses to Hypoxia during Infection by the Human Fungal Pathogen Cryptococcus neoformans
Source: PLoS Pathog. 2007 Feb 23;3(2):e22. doi: 10.1371/journal.ppat.0030022 (PMC1803011; doi:10.1371/journal.ppat.0030022)

FIGURE S1

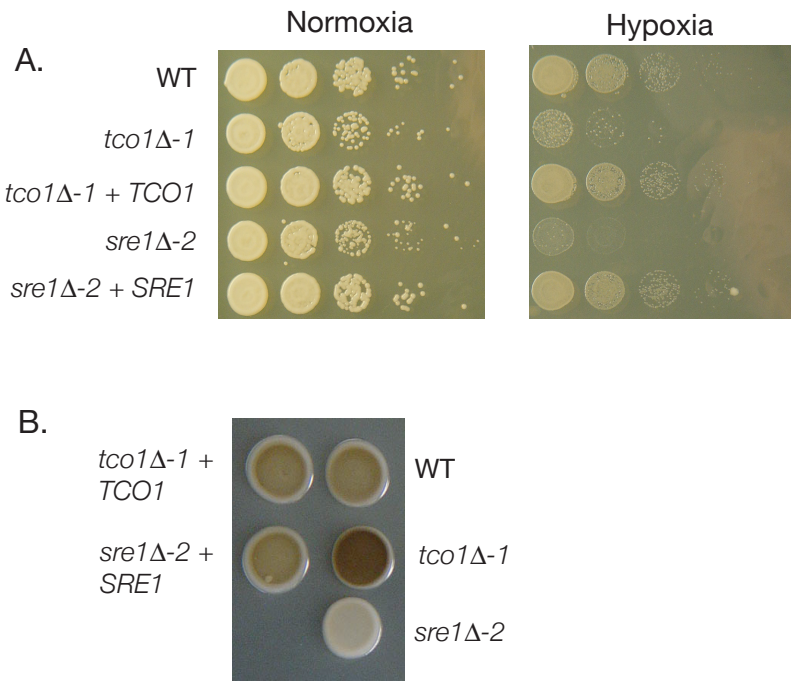

Supplement: Figure S1 — (A) SRE1 was re-introduced into its endogenous locus in sre1Δ-2 and TCO1 was re-introduced into its endogenous locus in tco1Δ-1. Cultures diluted to OD600nm = 0.6 were diluted serially in 10-fold increments prior to being spotted onto YPD plates. Plates were incubated in normoxic or hypoxic (controlled atmosphere chamber; less than 0.2% oxygen) conditions in the dark at 37 °C. (B) Melanin assays. The indicated strains were grown to saturation and spotted onto L-DOPA–containing medium. The plates were then cultured at 37 °C in the dark. (4.1 MB PDF) [file ppat.0030022.sg001.pdf]

FIGURE S2

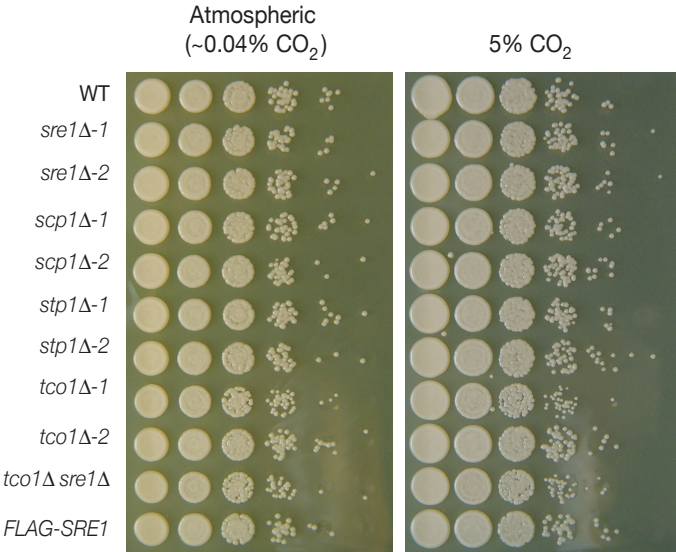

Supplement: Figure S2 — Cultures diluted to OD600nm = 0.6 were diluted serially in 10-fold increments prior to being spotted onto YPD plates. Plates were incubated in the dark at 37 °C in normal atmospheric conditions or in air supplemented with 5% CO2 (NuAire IR Autoflow CO2 Water-Jacketed Incubator, http://www.nuaire.com). (7.9 MB PDF) [file ppat.0030022.sg002.pdf]
