# Supplementary material for: A Link between Virulence and Homeostatic Responses to Hypoxia during Infection by the Human Fungal Pathogen Cryptococcus neoformans
Source: PLoS Pathog. 2007 Feb 23;3(2):e22. doi: 10.1371/journal.ppat.0030022 (PMC1803011; doi:10.1371/journal.ppat.0030022)
Supplement: Table S1 — (38 KB DOC) [file ppat.0030022.st001.doc]

# TABLE S1 – Strains used in this study

| **Strain Name** | **Genotype** | **Primer Set Used to Generate KO Construct** | **Parental Strain** |
| --- | --- | --- | --- |
| CM018 | H99 |  |  |
| CM092 | *sre1Δ-1::natR* | CN4363 | CM018 |
| CM093 | *sre1Δ-2::natR* | CDS_5817 | CM018 |
| CM094 | *scp1Δ-1::natR* | CN1329 | CM018 |
| CM095 | *scp1Δ-2::natR* | CDS_2877 | CM018 |
| CM096 | *stp1Δ-1::natR* | CDS_3547 | CM018 |
| CM097 | *stp1Δ-2::natR* | CDS_3547 | CM018 |
| CM098 | *tco1Δ-1::natR* | CN1538 | CM018 |
| CM099 | *tco1Δ-2::natR* | CDS_3015 | CM018 |
| CM100 | *tco1Δ-1::natR sre1Δ-2::neoR* | CDS_5817 | CM098 |
| CM101 | *FLAG-SRE1, natR* |  | CM018 |
| CM102 | *sxi1Δ::natR* | CN5693 | CM018 |
| CM103 | *lac1Δ::natR* | CN2897 | CM018 |
| CM111 | *SRE1* *neoR* |  | CM093 |
| CM112 | TCO1 neoR |  | CM098 |
