# Supplementary material for: A Link between Virulence and Homeostatic Responses to Hypoxia during Infection by the Human Fungal Pathogen Cryptococcus neoformans
Source: PLoS Pathog. 2007 Feb 23;3(2):e22. doi: 10.1371/journal.ppat.0030022 (PMC1803011; doi:10.1371/journal.ppat.0030022)
Supplement: Table S2 — (114 KB DOC) [file ppat.0030022.st002.doc]

# TABLE S2 – Primers used in this study

| **Primer Name** | **Used to create:** | Primer Sequence |
| --- | --- | --- |
| 5'-NAT-10 |  | CCGCTGCTAGGCGCGCCGTGAGCTGCGAGGATGTGAGCTGG |
| 3'-NAT-10 |  | GCAGGGATGCGGCCGCTGACAGAGCTCCACCGCGGTGGCGG |
| 3'-NEO-10 |  | GCAGGGATGCGGCCGCTGACAGAAGAGATGTAGAAACTAGCTT |
| VER-5-3 |  | GAATCCTGCATGCTTATGTG |
| VER-3-2 |  | CATGGCTCCTTGTCTCTGAA |
| CN4363-V5 | *sre1Δ-1* | TGATCTGTTCGGCCAGGGCGGAAA |
| CN4363-W1 | *sre1Δ-1* | AGAGAAGAAAGGCAACGCAAGGCT |
| CN4363-W3 | *sre1Δ-1* | CACGGCGCGCCTAGCAGCGGAAGGTGAAGCGGGCCTTGCCGCATG |
| CN4363-W4 | *sre1Δ-1* | GTCAGCGGCCGCATCCCTGCACGTCTTCTCTTTGACGATAAATTA |
| CN4363-W6 | *sre1Δ-1* | TCTTGCTCCTGCATTCTTTCTTCT |
| CN4363-V3 | *sre1Δ-1* | TGTTTTCGGGTAGTCTTTGAAAAC |
| CDS_5817-V5 | *sre1Δ-2* | TAGGATGCCCCGTCGTATTGTCTA |
| CDS_5817-W1 | *sre1Δ-2* | AGTAGAAGTAGTATAGTGGAATGG |
| CDS_5817-W3 | *sre1Δ-2* | CACGGCGCGCCTAGCAGCGGAGAATGTCGTCCTTATTTTCCGTCT |
| CDS_5817-W4 | *sre1Δ-2* | GTCAGCGGCCGCATCCCTGCACAGTGAGGTCGGCATCTTGTCCTG |
| CDS_5817-W6 | *sre1Δ-2* | AGCTGAGATTAAGCGTATGGAAGA |
| CDS_5817-V3 | *sre1Δ-2* | CTCGTGGCAAGGATATGGCTAACG |
| CN1329-V5 | *scp1Δ-1* | AAAGATTTCCTCCACCGAGCCCAC |
| CN1329-W1 | *scp1Δ-1* | TGGCCACCTTTGGACAATGATGCC |
| CN1329-W3 | *scp1Δ-1* | CACGGCGCGCCTAGCAGCGGAGTTGAACGATAAGAGTGGTGAAAA |
| CN1329-W4 | *scp1Δ-1* | GTCAGCGGCCGCATCCCTGCAAGGTCGGCATGGTTTCTCCGGTGG |
| CN1329-W6 | *scp1Δ-1* | ACACACGGCAACATGCTGACCATC |
| CN1329-V3 | *scp1Δ-1* | CCCTCTCTGGGCACTTCCCATATC |
| CDS_2788-V5 | *scp1Δ-2* | CCATGCTACCTTTTGTAGGTACGG |
| CDS_2788-W1 | *scp1Δ-2* | ATGATGACTGGTTGAGGTGGACTT |
| CDS_2788-W3 | *scp1Δ-2* | CACGGCGCGCCTAGCAGCGGATGCTCAAGTGGAGAATAAGTTGAC |
| CDS_2788-W4 | *scp1Δ-2* | GTCAGCGGCCGCATCCCTGCATGCTACCGGTCGTTCGGTACAGGA |
| CDS_2788-W6 | *scp1Δ-2* | GTCATCGAGACAGGGCTACTTAGC |
| CDS_2788-V3 | *scp1Δ-2* | CGAACAACGGATTGTTGCGCATCG |
| CDS_3547-V5 | *stp1Δ-1, stp1Δ-2* | TATTAGCGACCGAAGTATGTTCTT |
| CDS_3547-W1 | *stp1Δ-1, stp1Δ-2* | CAAGAATTCGGACGCCTTGAAGAT |
| CDS_3547-W3 | *stp1Δ-1, stp1Δ-2* | CACGGCGCGCCTAGCAGCGGAAAGCGTTTCACCACTTATTGGTTA |
| CDS_3547-W4 | *stp1Δ-1, stp1Δ-2* | GTCAGCGGCCGCATCCCTGCAATCTTGAGGGGGTCCGCTCTTGAG |
| CDS_3547-W6 | *stp1Δ-1, stp1Δ-2* | GTTGCGCCCCTTCAGATCTACGGA |
| CDS_3547-V3 | *stp1Δ-1, stp1Δ-2* | TGATATCATGACTTTCCGAGGGAA |
| CN1538-V5 | *tco1Δ-1* | TATGATTTAATACTTCGTGAACAA |
| CN1538-W1 | *tco1Δ-1* | TGGCCTTCTCCAAGCTTGAAGTGT |
| CN1538-W3 | *tco1Δ-1* | CACGGCGCGCCTAGCAGCGGAATTCAACTAGATGACGGCCTCCAT |
| CN1538-W4 | *tco1Δ-1* | GTCAGCGGCCGCATCCCTGCAACAGAAGCGTTGTCTTGCAGAGTG |
| CN1538-W6 | *tco1Δ-1* | AGAAAGGGTCGGGAGGCATTAATC |
| CN1538-V3 | *tco1Δ-1* | AGCAGGTCAATGGACAACATAGTA |
| CDS_3015-V5 | *tco1Δ-2* | GTTATGAAGTTTCTGTTGGCCGTA |
| CDS_3015-W1 | *tco1Δ-2* | CCAAAACACTGCGGATACGCGCAT |
| CDS_3015-W3 | *tco1Δ-2* | CACGGCGCGCCTAGCAGCGGAACCCTTCTCTCGTTCGCCCGATTG |
| CDS_3015-W4 | *tco1Δ-2* | GTCAGCGGCCGCATCCCTGCACGGCCAACCTTGCCCGTTTGTGGG |
| CDS_3015-W6 | *tco1Δ-2* | GAGACAGTATATAGGGCACGGACG |
| CDS_3015-V3 | *tco1Δ-2* | GTGTCAGGTGTCAAGTGTCAGGGT |
| CN5693-V5 | *sxi1Δ* | CGATGGAGCTAGAGGAAGGCGTAA |
| CN5693-W1 | *sxi1Δ* | TAGCTAATCGCTCCCCAAACCATC |
| CN5693-W3 | *sxi1Δ* | CACGGCGCGCCTAGCAGCGGATTAATACATATACACCATGCATCT |
| CN5693-W4 | *sxi1Δ* | GTCAGCGGCCGCATCCCTGCATCCAGAAGATCGTCCTGAAGAGAC |
| CN5693-W6 | *sxi1Δ* | AGCCATGGCTAAAAACCGTATGCA |
| CN5693-V3 | *sxi1Δ* | CTGACAATTTCTTCCCTGTAGACC |
| CN2892-V5 | *lac1Δ* | GGTTCACCTATGTCGTACTGCCAT |
| CN2897-W1 | *lac1Δ* | ACCGCAAAAGGAATTTAAGGTTTC |
| CN2897-W3 | *lac1Δ* | CACGGCGCGCCTAGCAGCGGATTAACTCACTTGCCAGTCTCCTCG |
| CN2897-W4 | *lac1Δ* | GTCAGCGGCCGCATCCCTGCAGAGTGACTATAGCTTGGATTGGAA |
| CN2897-W6 | *lac1Δ* | AGGCCTTTGATTTCGTTCGCTTGG |
| CN2897-V3 | *lac1Δ* | GTAATAAGCCCCTCCAAGCTTTTC |
| C_5817-V5FLe | FLAG-*SRE1* | ACGACACCTGCAGCAAATCTTTCC |
| C_5817-5Re | FLAG-*SRE1* | TTGGGCCTTCTTTCTCGAAACGAT |
| C_5817-5FgFl | FLAG-*SRE1* | GACGATGACAAGGGTAGTGGAAGCGGCTCTGCTGGCCCCACCCCTACCATTG |
| C_5817-FLAGRg | FLAG-*SRE1* | CCACTACCCTTGTCATCGTCATCTTTAT |
| C_5817-FLAGFg | FLAG-*SRE1* | GAAGCAGTTCCTGCTAGGGAACAAAAGCTGGAGCTCGAT |
| C_5817-W1FLg | FLAG-*SRE1* | TCCCTAGCAGGAACTGCTTCAGTTTTAAC |
| C_p5817-W3 | FLAG-*SRE1* | CACGGCGCGCCTAGCAGCGGAACATTTTACGATGATAGTATGAAC |
| C_CPR202-W4 | FLAG-*SRE1* | GTCAGCGGCCGCATCCCTGCAATTGGCACTTCCATGGTCCCTATC |
| C_CPR202-3F | FLAG-*SRE1* | CCCATGACCATATGTACGCTAATG |
| C_ACT1-1 |  | CTGTCTTCCCTTCTATTGTTGGTC |
| C_ACT1-2 |  | CTCAATGGGGTACTTCAAGGTAAG |
| C_ERG1-5-5 |  | ATCCTAAGAGAAGGGTGTTTCAAG |
| C_ERG1-3-5 |  | AATGGCGCCTACAGATCCGTTTGT |
| C_ERG3-5-3 |  | TCTCGGCCTATTCGTCTTTGTCCA |
| C_ERG3-3-5 |  | CGGGGTCACGATATGAGTCAAAGT |
| C_ERG5-5-1 |  | AGCTACCATCAAGGAAGTTTTGA |
| C_ERG5-3-1 |  | CGGGCTCAGGGTAACAGGTCTCGT |
| C_p5817-W1h |  | CCGCTGCTGTTGCTGCATTGGTTG |
| C_p3015-W1 |  | GCGGCCAACCTTGCCCGTTTGTGG |
| C_p3015-W3 |  | CACGGCGCGCCTAGCAGCGGACATCGGTACATTTTATGTGTAGTG |
| C_p3015-W4 |  | GTCAGCGGCCGCATCCCTGCAAACTGCATATATGTGAAGATAGCG |
| C_p3015-W6 |  | TAATCATATTCGACTTTCTTAGAT |
